# Effects of dietary inclusion of dry distillers grains with solubles on performance, carcass characteristics, and nitrogen metabolism in meat sheep: a meta-analysis

**DOI:** 10.3389/fvets.2023.1141068

**Published:** 2023-06-20

**Authors:** Sai Chandan Chelkapally, Thomas H. Terrill, Zaira M. Estrada-Reyes, Ibukun Michael Ogunade, Andres Alfredo Pech-Cervantes

**Affiliations:** ^1^Agricultural Research Station, Fort Valley State University, Fort Valley, GA, United States; ^2^Department of Animal Science, North Carolina A&T State University, Greensboro, NC, United States; ^3^Division of Animal and Nutritional Science, West Virginia University, Morgantown, WV, United States

**Keywords:** DDGS, sheep, performance, carcass, nitrogen efficiency

## Abstract

We conducted a meta-analysis in this scientific study to determine the effects of feeding meat sheep dry distillers grains with solubles (DDGS). Thirty-three peer-reviewed articles that met our inclusion requirements and were published between 1997 and 2021 were examined. To calculate the variation in performance, fermentation, carcass features, and nitrogen efficiency between the DDGS and control (no DDGS) treatments, we used 940 sheep weighing an average of 29.1 ± 1.5 kg. We used a hierarchical mixed model to conduct a meta-regression, subset, and dose–response analysis, while taking into consideration categorical variables like breed (pure or cross-breed), and continuous factors, like CP, NDF, and DDGS inclusion rate. Our findings indicate that sheep fed DDGS had higher (*p* < 0.05) final body weight (51.4 vs. 50.4 kg), neutral detergent fiber digestibility (55.9 vs. 53.8%), and total-tract ether extract digestibility (81.7 vs. 78.7%) than sheep on a control diet. No effects were observed on DMI, CP, and rumen fermentation, but dietary DDGS tended to increase (*p* = 0.07) HC weight (25.53 vs. 24.6 kg) and meat (redness) color (16.6 vs. 16.3) among treatment comparisons. Dietary DDGS was associated with higher N intake (29.9 vs. 26.8 g/d), fecal N (8.2 vs. 7.8 g/d), and digestibility (71.9 vs. 68.5%). Urinary nitrogen was significantly (*p* < 0.05) affected linearly by increasing the intake of DDGS in the diet. Based on the dose–response analysis, dietary DDGS inclusion should not exceed 20% to avoid negative effects on performance, nitrogen metabolism, and meat color. Dietary protein from DDGS should not exceed 17% to prevent reduced TVFA concentrations. Breed strongly influenced (*p* < 0.05) RMD in performance, and inconsistent responses were observed between crossbreed and purebred sheep comparisons. Despite these inconsistencies, no publication bias was observed, but a high variance (Ω^2^) among comparisons-between-studies was detected. This meta-analysis showed evidence in support of the hypothesis that feeding meat sheep DDGS at a rate of 20% can improve their performance, digestibility, carcass weight, and meat color.

## Introduction

In ruminant feeding systems, using agro-industrial byproducts, such as dry distillers grains with solubles (DDGS), has long been a practice. Dry distillers grains with solubles are the main byproduct of the manufacturing of ethanol from corn (1), and its popularity is growing around the world (2). Feed prices have increased, resulting in lower revenue and profit for farmers (3). Therefore, farmers are increasingly using alternative feeds, such as DDGS, in ruminant diets to reduce expenses (4).

Ethanol made from corn are widely used as fuel or for pharmaceutical and medical purposes (5). To obtain ethanol, the grain’s starch is fermented and eliminated. As it is the byproduct of making ethanol (6), over the last ten years, the yearly production of DDGS has increased from 20 million metric tons (MMT) to 41.56 MMT, more than doubling (7).

In accordance with NRC 2007, DDGS include more than 30% crude protein (CP), 73% of which is not digestible in the rumen, 40% NDF, and 11% fat (8). Also, the low nitrogen insoluble in acidic detergent (ADIN) content of DDGS reflects higher protein digestibility, and the higher PB2 + PB3 fraction of the Cornell net carbohydrate and protein system (CNCPS) fractionation system projects its use as a bypass protein source for animals (9). As a result, DDGS can serve as bypass proteins in ruminants when given at a dose of less than 150 g/kg DM, or as an energy source in ruminant diets when given at a dose of more than 150 g/kg DM (10). Because of its high calorie and fat content (3.67–4.34 Mcal/kg DM) (10), it is a highly digestible and economical feed component for ruminants (4).

Previous research on beef and dairy cattle shows that DDGS can improve their performance and growth when added to their diets at 50 and 21%, respectively (11, 12). Although numerous researchers have examined the impact of DDGS on sheep performance and growth (7, 13) the optimum amount of DDGS inclusion in sheep diets is still unknown. One of the possible risks of DDGS is the possibility of higher sulfur content in the diet, which can result in PEM in ruminants (1, 7). Determining the optimal DDGS inclusion amount in sheep diets is essential to avoiding negative effects on nitrogen efficiency and animal performance. Using a meta-analytic method, this research aims to determine the ideal inclusion amount of DDGS for sheep by assessing the effects of dietary supplementation with DDGS on sheep performance, fermentation, carcass features, and nitrogen efficiency.

## Materials and methods

### Literature search and inclusion criteria

Following the methodology described by Oliveira et al. (14) and Arriola et al. (15), a systematic search was carried out utilizing the databases ScienceDirect, Google Scholar, PubMed, The Web of Science, Scopus, and the Directory of Open Access Journals, to create a comprehensive database (2021). The search was conducted with the keywords DDGS, Sheep, Intake, Body weight (BW), Performance, Fermentation, Carcass features, and Nitrogen efficiency. Studies were only included if they met a set of requirements, which included being published in English peer-reviewed journals, focusing solely on sheep, reporting intake, body weight, and average daily gain, comparing DDGS with control, and reporting standard error of the mean (SEM), standard deviation (SD), and the number of experimental units per treatment. The final database only contained research published between 1997 and 2021 that satisfied the inclusion requirements, whereas excluded studies and duplicate records were removed. The initial database covered the period from 1985 to 2021.

### Data extraction

**Figure 1:**
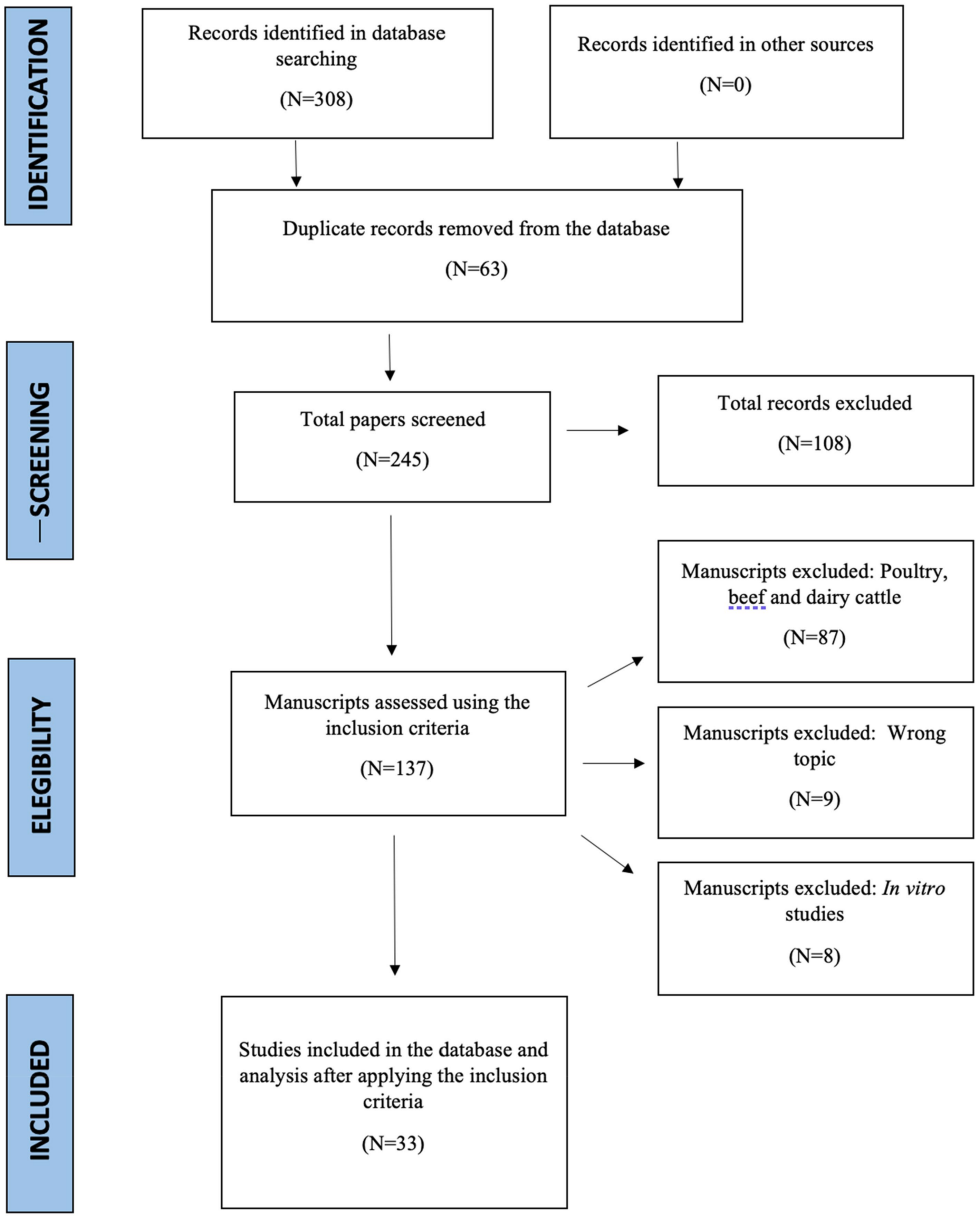
PRISMA workflow diagram from the meta-analysis of dietary supplementation of dry distillers grains with solubles (DDGS) on performance, fermentation, carcass characteristics, and nitrogen efficiency in sheep.

**Figure 2:**
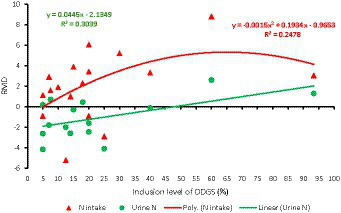
Dose–response plot of the dietary inclusion of dried distillers grains with solubles (DDGS) on nitrogen intake (NI) and urinary nitrogen (NU) in sheep. Raw mean difference (RMD) was performed between DDGS vs. control.

The PRISMA methodology was followed during the data extraction process, which is shown in Figure 1 (16). Three hundred eight peer-reviewed publications were incorporated into the database after the first screening. However, 275 manuscripts were excluded because they were thesis papers, lacked adequate data analysis and reporting (108 manuscripts), wrong topic (9 manuscripts), and *in vitro* research (8 manuscripts). Replicates, means, and SEM were extracted from the data relevant to the control and DDGS treatments. For each treatment, different response variables, including dry matter intake (DMI), total-tract digestibility of dry matter (DMD), total-tract crude protein digestibility (CPD), total-tract neutral detergent fiber digestibility (NDFD), total-tract ether extract digestibility (EED), initial body weight (IBW), final body weight (FBW), average daily gain (ADG), feed to gain ratio (F:G), hot carcass weight (HC), cold carcass weight (CC), dressing percentage, back fat, yield grade, muscle color lightness (MC *l*), muscle color redness (MC *a*), muscle color yellowness (MC *b*), rumen pH, total volatile fatty acids (TVFA), acetate, propionate, and butyrate molar proportions, acetate and propionate ratio, and ammonia (NH_3_-N), nitrogen intake, nitrogen urine loss, nitrogen fecal loss, nitrogen retention, and nitrogen digestibility were recorded. Moreover, the dry matter (DM) content, crude protein (CP), neutral detergent fiber (NDF), acid detergent fiber (ADF), ether extract (EE), sheep breed (Table 1), DDGS type, and DDGS concentration in the dietary treatments were noted and used as covariates. Based on inclusion criteria, 33 papers with 940 sheep (29.1 ± 1.5 kg) were assigned to 60 treatment comparisons, with DDGS inclusion rates in diets ranging from 0 to 100% (Figure 2).

**Table 1:** Summary of the breed and inclusion level of DDGS fed to sheep.

Type of breed	Breed	DDGS inclusion level (% DM)	Author
Crossbreed	Suffolk ^X^ Dorsett	0, 93.4	Archibeque et al. (17)
Crossbreed	Suffolk ^X^ Hampshire rams	0, 14.9, 29.7, 44.3	Crane et al. (18)
Crossbreed	Suffolk ^X^ Western whiteface	0, 15, 30	Van Emon et al. (19)
Crossbreed	Suffolk ^X^ Rambouillet	0, 15, 30	Crane et al. (20)
Crossbreed	Creole ^X^ Rambouillet	0, 15, 30, 45	Curzaynz-Leyva et al. (6)
Crossbreed	Pelibuey ^X^ Katahdin	0, 15, 30, 45	Castro-Perez et al. (21)
Crossbreed	Romanov ^X^ Rahmani male	0, 6, 9, 12	Gabr et al. (22)
Crossbreed	Whiteface ^X^ not reported	0, 30, 45	Lundy et al. (23)
Crossbreed	Not reported	0, 40	Lodge et al. (24)
Crossbreed	Not reported	0, 22.9	Huls et al. (25)
Pure breed	Awassi	0, 20, 30	Alshdaifat et al. (10)
		0, 7.5, 15	Obeidat et al. (26)
		0, 7, 14	Aloueedat et al. (27)
		0, 12.5, 25	Hatamleh et al. (5)
Pure breed	Rambouillet	0, 33, 66, 100	McEachern et al. (28)
		0, 20, 40, 60	Neville et al. (29)
		0, 20, 40, 60	Schauer et al. (30)
Pure breed	Canadian Arcott	0, 10, 30,47	Avila-Stagno et al. (31)
		0, 20	O’Hara et al. (32)
Pure breed	Merino	0, 20, 40	Graham et al. (33)
		0, 23.8, 91.1	Moyo et al. (34)
Pure breed	Hu	0, 20	Shen et al. (35)
		0, 5	Chen et al. (3)
Pure breed	Gulf coast	0, 12.7, 25.4	Abdelrahim et al. (36)
Pure breed	Katahdin	0, 10, 20, 30	Castro-Perez et al. (37)
Pure breed	Mexican Creole	0, 20, 40	Curzaynz-Leyva et al. (13)
Pure breed	Nellore	0, 50, 75, 100	Reddy et al. (7)
Pure breed	Lacaune	0, 18	De Evan et al. (38)
Pure breed	Wrzosowka ram	0, 45	Kawecka et al. (39)
Pure breed	Barki lambs	0,30,40, 50	Ghoneem et al. (8)
Pure breed	Tuj lambs	0, 10,20	Sahin et al. (40)
Pure breed	Whiteface	0,10, 15.34, 25.39	Zelinsky et al. (41)
Pure breed	Merino	0, 20, 40, 60	Felix et al. (42)

### Statistical analysis

Weighed raw mean differences (RMD) between the control and DDGS treatments were used to determine the effects of dietary inclusion on performance and rumen fermentation. In a hierarchical effects model with a robust variance estimation based on Tipton, the computed weighting used the inverse of variance (43). The heterogenicity was computed using *I*^2^, which Higgins (44) proposed. It is the ratio of the variance effects of the treatment divided by the overall variance observed (45). Also, the variance component between clusters (τ^2^) and between-studies-within-cluster (Ω^2^) were estimated using the procedure previously published by Hedges et al. (46) and Fisher et al. (47). Briefly, using the formula SD = SEM x $\sqrt{n}$ , where n is the number of experimental units, the pooled SD in each comparison within the study (DDGS vs. Control) was determined from the reported SEM. The overall effect size was then determined using the weighted RMD (SEM and n), *τ*^2^, and Ω^2^ statistics. Moreover, publication bias was calculated using the techniques outlined by Egger et al. (48) and implemented by Arriola et al. (15). Cook’s distances were calculated to remove outliers and significant points, and standardized residuals lower than 2.3 were regarded as acceptable (49).

**Figure 3:**
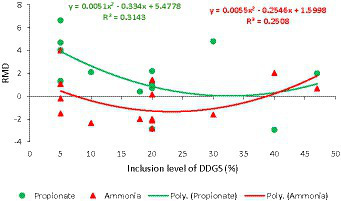
Dose–response plot of the dietary inclusion of dried distillers grains with solubles (DDGS) on the molar concentration of propionate and ammonia in the rumen of sheep. Raw mean difference (RMD) was performed between DDGS vs. control.

The meta-regression was carried out to determine the effects of the covariates, such as CP, Sheep breed (crossbred = 1 and pure breed = 2), and DDGS inclusion level in the diet, that influenced the effect size in the response variables (Table 2). Following the steps outlined by Viechtbauer et al. (50) and Oliveira et al. (14), the Wald test multiparameter technique was used to calculate the effects of the covariates on the model (2017). When the covariates were significant, Pech-Cervantes et al. (49) modified Greenland’s method (51, 52) and used it to calculate the dose–response and trend (2022). Egger’s regression method’s asymmetry test between RMD and SE was used to calculate and demonstrate publication bias using funnel plots (14, 48). Following the technique outlined by Arriola et al. (15), cook’s distances were also utilized to exclude outliers and influential points (Figure 3).

**Table 2:** Chemical composition and descriptive statistics of the experimental diets and treatments for sheep fed with dried distillers grains with solubles (DDGS).

Item	*N* ^1^	Mean	Std	Min	Max	Median
DM (% of as fed)	60	80.4	20.3	25.7	95.2	90.1
CP (% of DM)	60	17.5	5.1	10.1	39.1	16.0
NDF (% of DM)	60	31.3	13.3	11.7	91.7	29.2
ADF (% of DM)	60	15.2	6.1	3.9	32.4	14.1
EE (% of DM)	60	3.5	1.9	1.0	10.3	2.7
DDGS^2^ level (% DM)	60	21.1	22.6	0	100	15

^1^Total number of treatment means (control and DDGS treatments).

^2^Dried distillers grain with solubles (percent unit per kg of dry matter).

According to Fisher et al. (50, 53) and Viechtbauer et al., all data analyzes, including RMD, forest plot, and meta-regression analysis, were carried out using the robumeta (version 1.3.1093;^1^) and metafor (version 1.3.1093;^2^) packages in Rstudio (Version 1.25).

## Results

### Dietary composition, animal performance, and rumen fermentation

**Figure 4:**
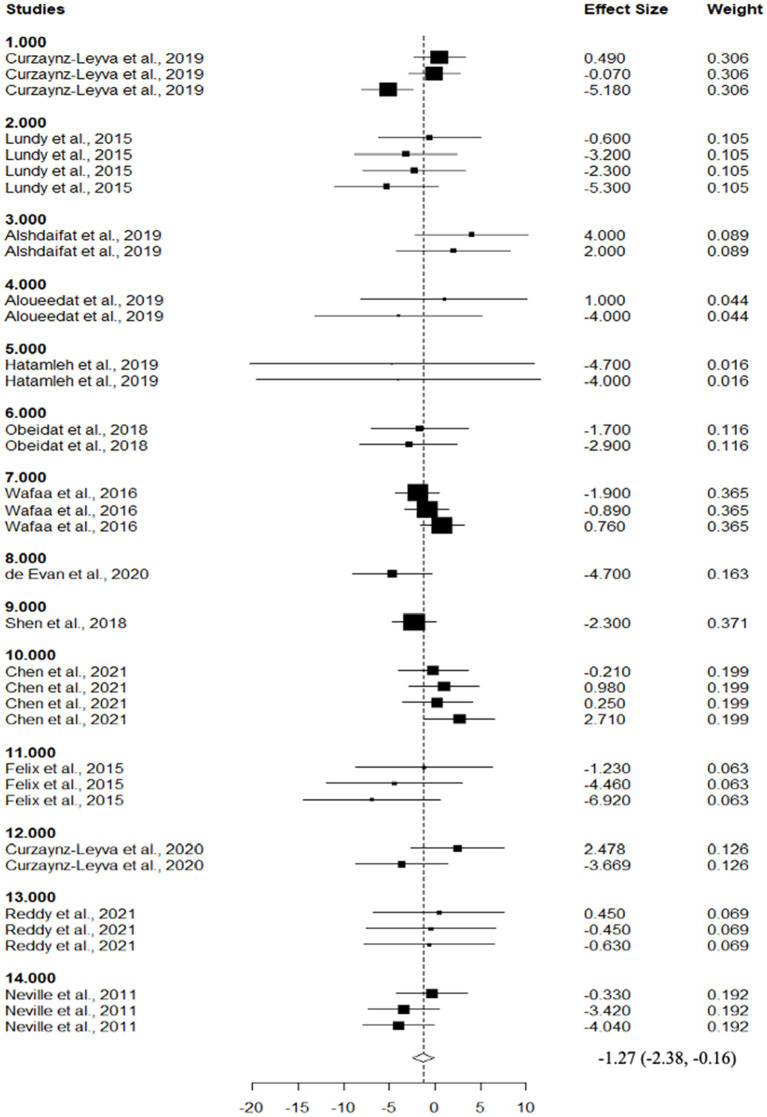
Forest plot of the effect of dietary inclusion of dried distillers grains with solubles (DDGS) on dry matter digestibility in sheep. The x-axis shows the RMD between DDGS and control, squares on the left indicate a decrease in DMD whereas squares on the right indicate an increase in DMD by DDGS. Lines connected to the squares correspond to the 95% confidence interval. The dotted vertical line represents the overall size effect estimate, and the diamond at the bottom represents the mean response across the studies.

Descriptive statistics are used in Table 2 to show the chemical composition of experimental diets that include various levels of DDGS. Despite the high heterogeneity found within studies (*p* > 0.05), the chemical composition was constant throughout literature comparisons, and no influential points were found to be present. Table 3 provides a summary of the effects of dietary DDGS on the performance and digestibility of sheep. Dietary DDGS had no significant affect on initial body weight, crude protein digestibility, feed efficiency, or intake of dry matter (DMI), as compared to the control (*p* > 0.05). However, feeding DDGS was associated with an increase in ether extract digestibility (EED) (*p* < 0.05), neutral detergent fiber digestibility (NDFD) (*p* < 0.05), and final body weight (FBW) (*p* < 0.05), and showed a tendency to increase the average daily gain (ADG) (*p* = 0.06). Comparisons showed that DDGS reduced dry matter digestibility (DMD) (*p* = 0.02) in comparison to the control (Figure 4). Dry matter intake (DMI) and digestibility showed low to moderate Ω^2^ values according to variance analysis, whereas ADG and BW had greater Ω^2^ values across comparisons. Furthermore, the funnel test revealed that most response factors had high heterogeneity (*I^2^* > 70%) for DMI, DMD, CPD, and F: G. Nevertheless, there was no significant publication bias among comparisons (*p* > 0.05).

**Table 3:** Effect of dietary supplementation of DDGS on intake, digestibility, and performance by sheep.

	Control^2^			RMD^3^		Variance component^14^		Bias	
Item	*N* ^1^	Mean	STD	Effect size	*p*-value^4^	Ω^2^	*τ* ^2^	Funnel test^6^ (*p-*value)	*I^2^* (%)
DMI^5^ (kg/d)	56	1.51	0.4	0.04 (−0.01,0.09)	0.11	0.001	0.003	0.30	72.77
DMD^6^ (%)	38	74.9	4.2	−1.27 (−2.38, −0.16)	0.02	1.21	0	0.47	84.75
CPD^7^ (%)	18	73.9	4.9	0.35 (−1.97,2.68)	0.36	4.01	0	0.45	82.10
NDFD^8^ (%)	34	53.8	11.2	2.13 (1.36,2.9)	<0.01	2.66	0	0.69	48.79
EED^9^ (%)	16	78.7	7.6	3.17 (2.14,4.2)	<0.01	0	0	0.70	34.16
IBW^10^ (kg)	57	29.1	11.5	0.07 (−0.24,0.34)	0.56	0.2	0	0.45	0
FBW^11^ (Kg)	57	50.4	18.2	1.03 (0.29,1.77)	<0.01	26.8	0	0.65	54.54
ADG^12^ (g/d)	60	247.6	92.8	6.47 (−0.4,13.4)	0.06	2027.2	0	0.86	54.93
F: G^13^	45	6.11	2.6	−0.03 (−0.25,0.20)	0.81	1.01	0	0.16	99.96

Positive values in RMD indicate an increase by the addition of DDGS, whereas negative values in RMD indicate a decrease by the addition of DDGS with respect to the control.

^1^Total number of comparisons.

^2^No DDGS in the diet.

^3^Raw mean difference between control vs DDGS treatment.

^4^*p*-value for *X*^2^ (Q) test for heterogeneity; *I*^2^, Proportion of total variation of size effect estimated due to heterogeneity.

^5^Total-tract dry matter intake.

^6^Total-tract dry matter digestibility.

^7^Total-tract crude protein digestibility.

^8^Total-tract neutral detergent fiber digestibility.

^9^Total-tract ether extract digestibility.

^10^Initial body weight.

^11^Final body weight.

^12^Average body gain.

^13^Feed to gain ratio.

^14^Ω^2^, between-studies-within-cluster variance component; *τ*^2^, between-cluster variance component (46, 47).

The effects of dietary DDGS on rumen pH and fermentation in meat sheep are shown in Table 4. Dietary inclusion of DDGS did not influence (*p* > 0.05) rumen pH (6.23 vs. 6.16), Total VFA (88.54 vs. 89.6 mmol/L), and propionate molar proportions (31.49 vs. 29.8%) among treatment comparisons (DDGS vs. control). However, dietary DDGS tended to decrease (*p* = 0.08) molar proportions of acetate (50.06 vs. 52%) and butyrate (11.08 vs. 11.9%). Conversely, dietary DDGS did not affect the ruminal concentration of NH_3_-N (19.1 vs. 18.1 mg/dL). However, dietary DDGS was associated with a lower (*p* < 0.05) A:P ratio (1.26 vs. 1.87) for DDGS treatment than control sheep. The variance analysis showed a lower Ω^2^ for the molar proportions of acetate, propionate, and butyrate between comparisons, and a larger Ω^2^ for total VFA concentrations. Rumen fermentation data exhibited high heterogeneity (*I^2^* > 70%) for pH and NH3-N, similar to animal performance data. Despite the high heterogeneity, there was no significant potential for bias between comparisons for pH and rumen fermentation. Furthermore, acetate and the A:P ratio showed significant bias when compared across treatments.

**Table 4:** Effect of dietary supplementation of DDGS on rumen pH, volatile fatty acids, and ammonia nitrogen concentrations of sheep.

	Control^2^			RMD^3^		Variance component^7^		Bias	
Item	*N* ^1^	Mean	STD	Effect size	*P*-value^4^	Ω^2^	*τ* ^2^	Funnel test^6^ (*p-*value)	*I^2^* (%)
pH	20	6.16	0.43	0.07 (−0.03,0.16)	0.14	0.006	0	0.44	79.26
TotalVFA^5^ (mmol/L)	17	89.6	33.3	−1.06 (−6.92,4.8)	0.62	257.1	0	0.34	42.46
Acetate (%)	14	52.0	5.5	−1.94 (−4.39,0.51)	0.08	0	0	0.04	49.66
Propionate (%)	14	29.8	5.8	1.69 (−1.37,4.75)	0.18	0	0	0.13	62.28
Butyrate (%)	14	11.9	1.6	−0.82 (−1.81, 0.16)	0.07	0	0	0.16	47.99
A: P^6^ ratio	14	1.87	0.61	−0.24 (−0.38, −0.09)	0.02	0	0	0.01	42.62
NH_3_-N (mg/dL)	14	18.1	10.9	0.09 (−2.21, 2.39)	0.91	20.17	0	0.61	87.41

Positive values in RMD indicate an increase by the addition of DDGS, whereas negative values in RMD indicate a decrease by the addition of DDGS with respect to the control.

^1^Total number of comparisons.

^2^No DDGS in the diet.

^3^Raw mean difference between control vs DDGS treatment.

^4^*p*-value for *X*^2^ (Q) test for heterogeneity; *I*^2^, Proportion of total variation of size effect estimated due to heterogeneity.

^5^Total volatile fatty acids.

^6^Acetate: Propionate ratio.

^7^Ω^2^, between-studies-within-cluster variance component; *τ*^2^, between-cluster variance component (46, 47).

### Carcass characteristics

**Figure 5:**
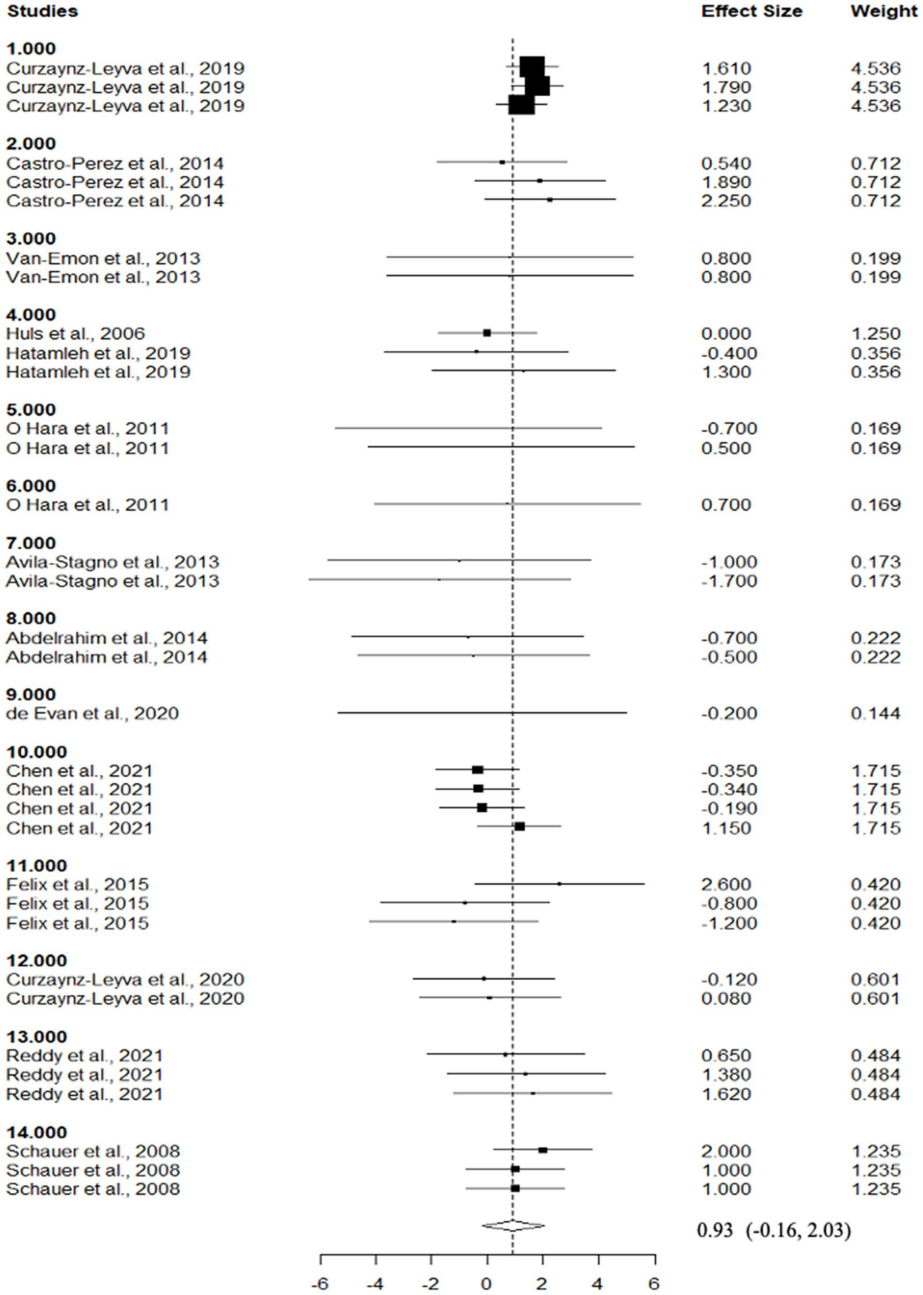
Forest plot of the effect of dietary inclusion of dried distillers grains with solubles (DDGS) on hot carcass weight in sheep. The x-axis shows the RMD between DDGS and control, squares on the left indicate a decrease in HCW whereas squares on the right indicate an increase in HCW by DDGS. Lines connected to the squares correspond to the 95% confidence interval. The dotted vertical line represents the overall size effect estimate, and the diamond at the bottom represents the mean response across the studies.

Table 5 illustrates the impact of dietary DDGS on the sheep carcass characteristics. Dietary supplementation with DDGS had no significant effect (*p* > 0.05) on dressing percentage (51.03 vs. 50.8%), MC *l* (41.29 vs. 40.7), and MC *b* (9.53 vs. 9.50) when compared to the control. Similarly, dietary DDGS did not affect (*p* > 0.05) back fat (0.46 vs. 0.47 cm) and yield grade (2.3 vs. 2.30) treatment comparisons. In contrast, dietary DDGS showed a trend to increase (*p* < 0.07) HC (25.53 vs. 24.6 kg; Figure 5), CC (21.1 vs. 19.8 kg), and (*p* < 0.08) MC *a* (16.59 vs. 16.3) relative to the control across comparisons. All carcass features among comparisons had a low Ω^2^ according to the variance analysis. Apart from yield grade, MC a, and back fat, most response variables showed higher heterogeneity (*I^2^* > 50%). Despite this high level of heterogeneity, no significant potential for bias was found for HC, dressing %, back fat, yield grade, MC l, or MC a; however, substantial potential for bias was found (*p* < 0.01) for CC and MC b.

**Table 5:** Effect of dietary supplementation of DDGS on hot carcass, cold carcass, dressing, back fat, yield grade, muscle color (l), muscle color (a), muscle color (b) of sheep.

	Control^2^			RMD^3^		Variance component^10^		Bias	
Item	*N* ^1^	Mean	STD	Effect size	*P*-value^4^	Ω^2^	*τ* ^2^	Funnel test^6^ (*p-*value)	*I^2^* (%)
HC^5^ (kg)	34	24.6	7.7	0.93 (−0.16, 2.03)	0.07	0	0	0.47	76.17
CC^6^ (kg)	14	19.8	6.9	1.31 (−0.87,3.49)	0.10	0	0	<0.01	68.50
Dressing (%)	20	50.8	4.8	0.23 (−0.41,0.86)	0.40	0	0	0.75	59.93
Back Fat (cm)	17	0.47	0.2	−0.01 (−0.05,0.04)	0.80	0	0	0.49	34.66
Yield grade	24	2.30	1.2	−0.005 (−0.21,0.22)	0.95	1.62	0	0.71	15.88
MC *l*^7^	8	40.7	5.4	0.59 (−2.1,3.3)	0.43	1.41	0	0.93	71.70
MC *a*^8^	8	16.3	9.0	0.29 (−0.12,0.71)	0.08	0.04	0	0.67	31.83
MC *b*^9^	8	9.5	6.5	0.03 (−1.69,1.75)	0.94	0	0.30	<0.01	80.58

Positive values in RMD indicate an increase by the addition of DDGS, whereas negative values in RMD indicate a decrease by the addition of DDGS with respect to the control.

^1^Total number of comparisons.

^2^No DDGS in the diet.

^3^Raw mean difference between control vs DDGS treatment.

^4^*p*-value for *X*^2^ (Q) test for heterogeneity; *I*^2^, Proportion of total variation of size effect estimated due to heterogeneity.

^5^Hot carcass.

^6^Cold carcass.

^7^Muscle color l (lightness).

^8^Muscle color a (redness).

^9^Muscle color b (yellowness).

^10^Ω^2^, between-studies-within-cluster variance component; *τ*^2^, between-cluster variance component (46, 47).

### Nitrogen metabolism and efficiency

Table 6 illustrates the impact of dietary DDGS on sheep’s nitrogen utilization. For the control and DDGS-supplemented groups across treatment comparisons, no significant differences (*p* > 0.05) were observed in NU (10.11 vs. 11.1 g/d) or NR (11.98 vs. 11.2 g/d). However, across treatment comparisons, dietary DDGS supplementation was correlated with higher (*p* < 0.05) NF (8.22 vs. 7.8 g/d) and had a tendency (*p* = 0.06) to increase NI (29.93 vs. 26.8 g/d) and ND (71.92 vs. 68.5%). The variance analysis indicates that the nitrogen efficiency across comparisons had a low Ω^2^ value. The funnel tests showed that, except for NU, the majority of response variables showed moderate heterogeneity (*I^2^* > 50%). Despite the high heterogeneity, no significant potential for bias was found across comparisons.

**Table 6:** Effect of dietary supplementation of DDGS on nitrogen intake, nitrogen urine output, nitrogen feces output, nitrogen retention, nitrogen digestibility of sheep.

	Control^2^			RMD^3^		Variance component^10^		Bias	
Item	*N* ^1^	Mean	STD	Effect size	*P*-value^4^	Ω^2^	*τ* ^2^	Funnel test^6^ (*p-*value)	*I^2^* (%)
NI^5^ (g/d)	18	26.8	8.7	3.13 (−0.30,6.56)	0.06	0	0	0.29	65.08
NU^6^ (g/d)	15	11.1	3.9	−0.99 (−2.66,0.67)	0.19	0	0	0.17	76.21
NF^7^ (g/d)	19	7.8	2.9	0.42 (0.02, 0.82)	0.04	0	0	0.60	52.18
NR^8^ (g/d)	15	11.2	4.5	0.78 (−0.24,1.81)	0.11	0	0	0.83	33.47
ND^9^ (%)	8	68.5	9.4	3.42 (−1.13,7.98)	0.07	0	0	0.46	40.85

Positive values in RMD indicate an increase by the addition of DDGS, whereas negative values in RMD indicate a decrease by the addition of DDGS with respect to the control.

^1^Total number of comparisons.

^2^No DDGS in the diet.

^3^Raw mean difference between control vs DDGS treatment.

^4^*p*-value for *X*^2^ (Q) test for heterogeneity; *I*^2^, Proportion of total variation of size effect estimated due to heterogeneity.

^5^Nitrogen intake.

^6^Nitrogen urine.

^7^Nitrogen feces.

^8^Nitrogen retention.

^9^Nitrogen digestibility.

^10^Ω^2^, between-studies-within-cluster variance component; *τ*^2^, between-cluster variance component (46, 47).

### Meta-regression, subset analysis, and dose–response analysis

**Figure 6:**
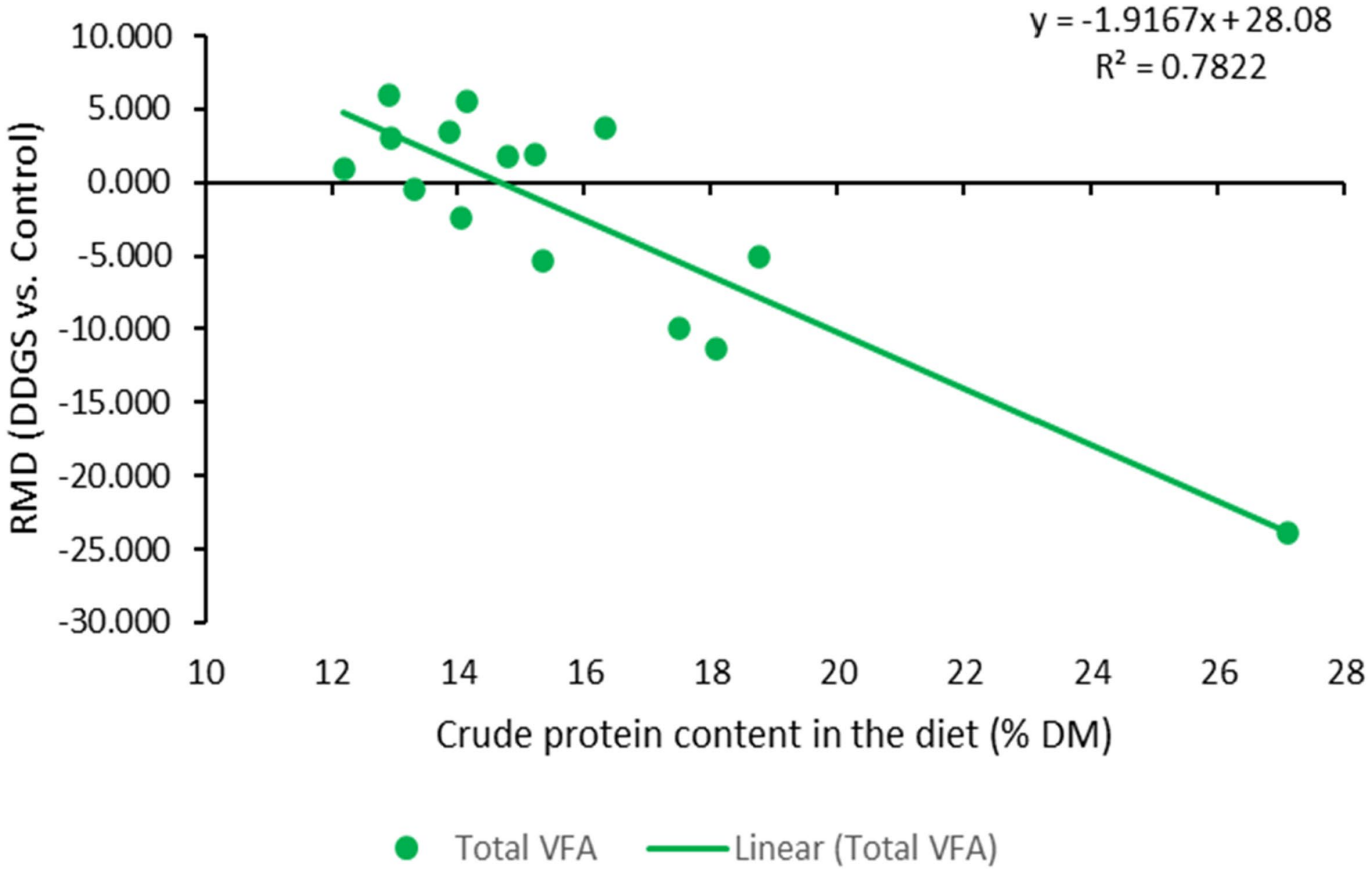
Dose–response plot of the dietary inclusion of dried distillers grains with solubles (DDGS) on crude protein content in the diet and total VFA in the rumen of sheep. Raw mean difference (RMD) was performed between DDGS vs. control.

**Figure 7:**
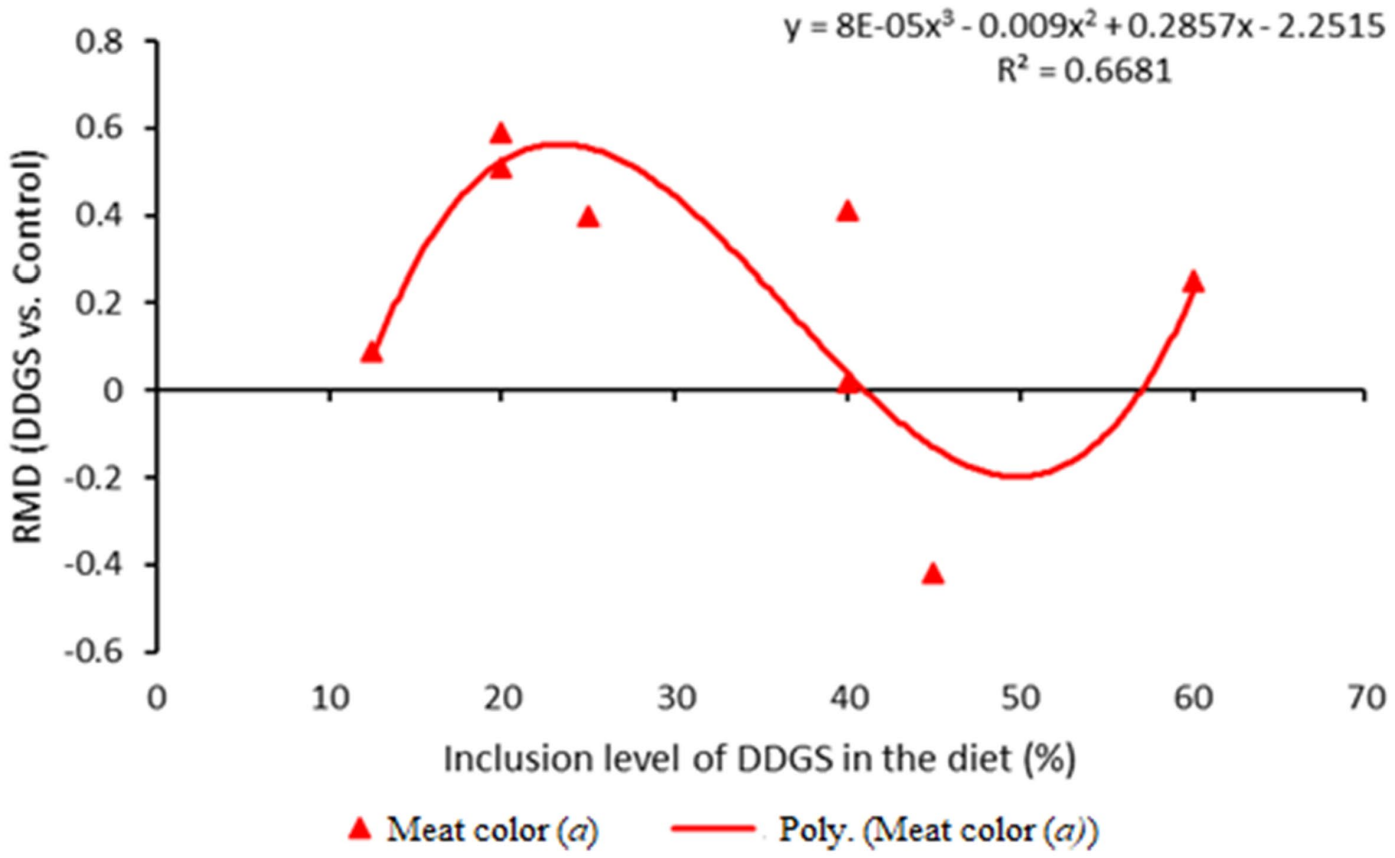
Dose–response plot of the dietary inclusion of dried distillers grains with solubles (DDGS) on meat color redness (*a*) in sheep. Raw mean difference (RMD) was performed between DDGS vs. control.

**Figure 8:**
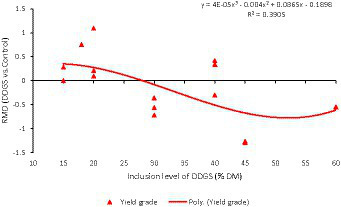
Dose–response plot of the dietary inclusion of dried distillers grains with solubles (DDGS) on yield grade in sheep. Raw mean difference (RMD) was performed between DDGS vs. control.

The results of the meta-regression analysis on the effects of dietary DDGS application on sheep performance, digestibility, rumen fermentation, carcass characteristics, and nitrogen efficiency are presented in Table 7. Covariates had no impact on the various intercepts (*p* > 0.05), but they reduced the Ω^2^ for all response variables in the meta-regression. Importantly, significant intercepts were seen for FBW, ADG, MC, and total VFA. Among all the covariates, breed strongly influenced (*p* < 0.05) the responses observed in FBW, ADG, HC, CC, and yield grade. Furthermore, breed tended to influence (*p* = 0.06) MC b among comparisons. Similarly, the dietary level of DDGS influenced (*p* < 0.05) yield grade, HC, and tended to influence (*p* = 0.08) ND among comparisons. The level of dietary CP significantly (*p* < 0.05) influenced the responses in FBW, HC, TVFA, and A:P, and it exhibited a tendency to influence (*p* = 0.06) ADG, (*p* = 0.07) DMI, and ND among treatment comparisons. Moreover, dietary NDF did not influence the responses observed in animal performance, digestibility, carcass, and rumen fermentation, but tended to influence (*p* = 0.06) the responses in ND among comparisons. The dose–response analysis demonstrated that the dietary level of DDGS should not be more than 20% to prevent negative impacts on nitrogen intake and urinary nitrogen (Figure 6), propionate molar ratios, and NH3-N concentrations in the rumen (Figure 7). Furthermore, overall VFA concentrations in the rumen reduced linearly (Figure 6; *R*^2^ = 78%) when the CP level in the meal increased (primarily impacted by DDGS inclusion level). Also, the dose–response analysis indicated that a 20% inclusion level in the diet of DDGS increased MC *a* (Figure 7; cubic effect), and yield grade (Figure 8; cubic effect).

**Table 7:** Meta-regression of the effect of dietary nutrient concentrations and dietary dose of dried distillers grain with solubles (DDGS) on raw mean differences (RMD) for performance, digestibility, carcass, rumen fermentation, and nitrogen efficiency of sheep.

Dependent variable	*N* ^1^	Intercept	*p*-value	DDGS^2^	*P*-value	CP^3^	*P*-value	NDF^4^	*P*-value	Breed	*P*-value	Ω^2^	*τ* ^2^
DMI^5^ (kg/d)	44	0.54	0.16	0.0005	0.46	−0.01	0.07	0.0009	0.71	−0.21	0.11	0.01	0
DMD^6^ (%)	35	2.19	0.63	−0.04	0.34	−0.14	0.45	−0.009	0.91	0.11	0.94	0	0.05
NDFD^7^ (%)	34	−1.28	0.82	−0.01	0.73	−0.02	0.80	−0.01	0.85	2.27	0.45	2.88	0
CPD^8^ (%)	17	−8.98	0.48	0.03	0.71	0.37	0.5	0.11	0.44	−0.31	0.89	2.63	0
EED^9^ (%)	16	−1.30	0.87	0.002	0.97	−0.004	0.97	0.26	0.42	−3.66	0.19	0	0
IBW^10^(kg)	45	−0.54	0.54	0.004	0.36	0.04	0.41	−0.02	0.28	0.19	0.50	0	0
FBW^11^(kg)	45	9.41	0.02	0.02	0.18	−0.26	0.04	−0.03	0.49	−2.23	<0.01	0	0
ADG^12^ (g/d)	48	90.1	0.01	0.08	0.60	−3.04	0.06	0.11	0.79	−23.13	<0.01	1528.9	0
F:G^13^	35	−0.03	0.97	−0.002	0.76	0.03	0.53	0.007	0.61	−0.38	0.18	3.03	0
HC^14^ (kg)	30	5.56	0.01	0.01	0.07	−0.14	0.03	−0.02	0.11	−1.47	<0.01	0	0
CC^15^ (kg)	13	8.43	0.10	0.05	0.15	−0.27	0.22	−0.08	0.16	−1.38	0.03	0	0
Dressing (%)	19	3.45	0.05	0.009	0.50	−0.22	0.11	0.06	0.11	−0.82	0.15	0	0
Back Fat (cm)	13	−0.11	0.55	−0.001	0.53	0.003	0.72	0.002	0.33	−0.001	0.99	0	0
Yield grade	16	−0.87	0.30	−0.03	0.02	0.05	0.20	−0.003	0.78	0.65	0.01	1.34	0
MC l^16^	7	−8.66	−0.05	0.05	0.11	0.43	0.04	0.003	0.95	2.07	0.10	0	0
MC a^17^	7	0.09	0.94	0.01	0.46	−0.14	0.45	0.06	0.24	0.51	0.25	0	0
MC b^18^	7	−5.75	0.02	−0.04	0.30	0.26	0.36	−0.03	0.64	1.97	0.06	0	0
pH	18	−0.33	0.07	0.001	0.35	0.01	0.16	0.007	0.10	0.001	0.56	0	0
Total VFA^19^ (mmol/L)	15	42.68	0.03	−0.04	0.58	−2.11	0.03	−0.29	0.43	−0.80	0.70	0	0
Acetate (%)	14	1.45	0.66	0.08	0.15	−0.02	0.86	−0.21	0.16	1.19	0.38	0	0
Propionate (%)	14	5.21	0.43	0.13	0.36	0.10	0.41	0.0009	0.99	−1.58	0.61	0	0
Butyrate (%)	14	4.47	0.32	0.02	0.30	−0.12	0.31	−0.14	0.26	0.73	0.70	0	0
A: P^20^ratio	14	0.29	0.37	0.009	0.33	−0.01	0.05	−0.02	0.23	0.16	0.45	0	0
NI^21^ (g/d)	17	7.46	0.38	0.20	0.06	−0.24	0.40	−0.11	0.39	−1.01	0.71	0	0
NU^22^ (g/d)	14	−10.8	0.42	0.02	0.83	0.55	0.49	−0.06	0.48	1.46	0.33	0	0
NF^23^(g/d)	18	0.09	0.98	0.02	0.67	0.07	0.81	−0.02	0.58	−0.19	0.82	0	0
NR^24^(g/d)	14	3.16	0.51	−0.02	0.54	0.08	0.75	−0.02	0.66	−1.61	0.17	0	0
ND^25^(%)	6	−44.5	0.06	−0.62	0.08	3.60	0.07	0.68	0.06	−2.87	0.14	0	0.01

Comparisons were performed between DDGS vs. Control.

^1^Number of comparisons.

^2^Dietary level of dried distillers grains with solubles (%).

^3^Crude protein in the diet.

^4^Neutral detergent fiber in the diet.

^5^Total-tract dry matter intake.

^6^Total-tract dry matter digestibility.

^7^Total-tract neutral detergent fiber digestibility.

^8^Total-tract crude protein digestibility.

^9^Total-tract ether extract digestibility.

^10^Initial body weight.

^11^Final body weight.

^12^Average body gain.

^13^Feed to gain ratio.

^14^Hot carcass.

^15^Cold carcass.

^16^Muscle color l (lightness).

^17^Muscle color a (redness).

^18^Muscle color b (yellowness).

^19^Total volatile fatty acids.

^20^Acetate: Propionate ratio.

^21^Nitrogen intake.

^22^Nitrogen urine.

^23^Nitrogen feces.

^24^Nitrogen retention.

^25^Nitrogen digestibility.

Ω^2^, between-studies-within-cluster variance component (46, 47).

τ^2^, between-cluster variance component (46, 47).

**Figure 9:**
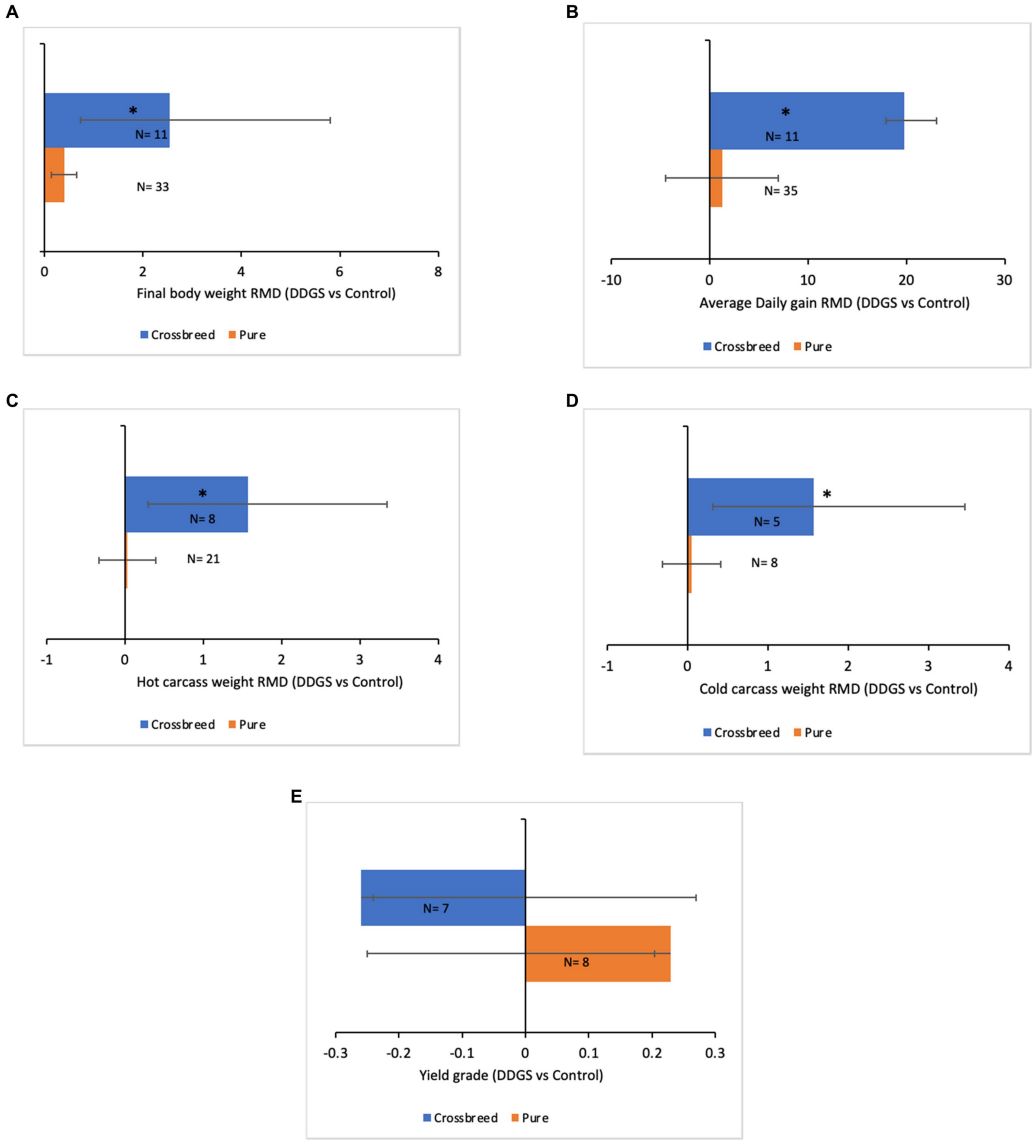
Subset analysis of the effect of breed (Pure or Crossbreed) on **(A)** Final body weight, **(B)** Average daily gain, **(C)** Hot carcass weight, **(D)** Cold carcass weight, and **(E)** Yield grade of meat sheep fed with dried distillers grains solubles. The effect size (weighted raw mean difference) was calculated in each comparison (DDGS vs. Control). Error bars represent the 95% confidence interval. The symbol (*) indicates significant difference (*p* < 0.05).

The subset analysis revealed that performance and carcass characteristics were strongly influenced by sheep breed (Figure 9). Thus, dietary DDGS increased FBW, ADG, HC, and CC in crossbreed studies compared to studies conducted with pure breeds. Moreover, dietary DDGS decreased yield grade in crossbreed studies compared to studies with pure breed sheep.

## Discussion

Factors like grain quality, milling procedure, fermentation process, and temperature have an effect on the quality and chemical composition of DDGS (36). The results of this meta-analysis suggest that the chemical composition of DDGS varied between comparisons in this aspect. According to earlier research (21, 37), the amount of solubles added during processing, as well as variations in distillers solubles proportions from plant to plant, have an effect on the lipid content and crude protein concentration in DDGS. However, the current meta-analysis suggests that animal responses were consistent across studies because these variations did not raise the publication bias (*I* value). Moreover, some of the variations in animal performance, rumen fermentation, and carcass characteristics were explained by the covariates used in this meta-analysis. The novelty of this study is in the relationship between the dietary inclusion of DDGS in the diet and meat quality of sheep, and additionally, it’s showing of a relationship between nitrogen metabolism, rumen fermentation, and carcass characteristics in meat sheep, which have not been shown in other studies. This study evaluates the effects of DDGS on nutritional, physiological, biochemical, and food science variables and uses a more comprehensive search strategy, a larger sample size, or more rigorous inclusion criteria in comparison to previous studies.

According to previous research, adding DDGS to sheep diets improves animal performance and carcass qualities (13, 54) by raising ADG and final body weight in meat sheep (4, 7). Collectively, DDGS supplementation had no effect on DMI or F: G ratio. In agreement with these results, a prior meta-analysis demonstrated that feeding DDGS to beef cattle increased their ADG and FBW (11). In addition, the current meta-analysis shows that included dietary DDGS increased FBW by 2.04% in comparison to the control. Likewise, improvements in performance and carcass weight in DDGS diets in this meta-analysis could be associated with the greater total-tract NDF and EE digestibility observed among treatment comparisons (7, 13). Previous research (34) has shown that inclusion of DDGS in the diet increased NDF digestibility. This meta-analysis showed that dietary DDGS inclusion increased NDFD and EED by 3.17 and 2.13%, respectively. The increased EED may result from the higher dietary EE content (5). However, DDGS supplementation reduced DMD without changing CP digestibility. The decrease in DMD may be due to the EE level in the diet exceeding the minimum threshold, which prevents microbes from adhering to the rumen digesta (5, 42). Previous research reported that dietary levels of DDGS above 20% linearly reduced total-tract DMD and total-tract starch digestibility, but increased total-tract NDF digestibility (55). The results of this meta-analysis may illustrate the impacts that have been noticed. The effect of DDGS on starch digestibility cannot be determined because of a shortage of data, but it is expected that in most comparisons, dietary DDGS decreased total-tract starch digestibility. However, more studies are required to confirm these hypotheses. Furthermore, this meta-analysis shows that DDGS improved performance (15 to 20% undegradable protein consumption and 8 to 12% fat) by providing bypass protein and fat to raise ultimate body weight (36). However, the dose–response study recommends that dietary DDGS not exceed 20% (DM basis) to prevent negative effects on total-tract digestibility, body weight, and carcass weight in meat sheep. The inconsistencies observed between comparisons suggest a different productive response to DDGS supplementation in crossbreed animals compared to purebred animals. These results could help to explain the high variance observed (Ω^2^) among comparisons-between-studies. The observed differences and high variance could be associated with the unaccounted effect of genotype x environment interaction, that has been known to influence animal performance (56), but was not estimated in this meta-analysis due to the limited amount of data. For instance, sheep breeds that are raised in cooler climates may have a higher ADG due to a decrease in heat stress, whereas those raised in warmer environments may require additional nutritional support to maintain optimal growth rates. Future studies should evaluate how dietary and genetic factors influence the response of DDGS in ruminants. The high level of crude protein in DDGS diets impacted fiber digestibility by increasing proteolysis and the production of NH_3_-N in the rumen (10). According to Li et al. (57), fermentation procedures or granular starch hydrolysis resulted in up to 18% of the starch in DDGS to escape digestion. Resistant starch altered the process by which nutrients and fibers from other sources in the diet were digested and fermented (58). One of the possible phenomena is that when animals consume DDGS feed that has a lot of resistant starch, the fermentation of that starch can produce short chain fatty acids and lower the pH, which leads to an acidic environment that is less hospitable to bacteria that break down fiber. As a result, there may be fewer microbes that break down fiber in the large intestine, which could eventually lead to a reduction of overall fiber digestibility in the feed (59). Among treatment comparisons, the molar ratios of acetate and the acetate: propionate ratio decreased as DDGS inclusion increased. Although conflicting results have been reported in the literature, the outcomes of our meta-analysis suggest that the excess bypass protein in DDGS was connected to decreased rumen fermentation (3, 31). Also, the dose–response analysis supports the hypothesis that dietary DDGS in meat sheep should not exceed 20% to prevent negative effects on rumen fermentation. Dietary inclusion of DDGS in the diet above 35% reduced propionate molar proportions and increased NH_3_-N concentration in the rumen. These results imply that the rumen microbiome is sensitive to level of bypass protein in the rumen (26). As a result, more research is required to determine how DDGS affects the rumen microbiome-metabolome interplay and its relationship to performance, fermentation, and meat quality in meat sheep.

According to earlier research (2, 4), adding DDGS up to 60% to sheep diets enhanced hot carcass weight (HC) and cold carcass weight (CC) without affecting dressing percentage or back fat. Previous research reported that bypass essential amino acids and fat from DDGS contributed to a greater extent to the net energy of lactation in dairy cows (60). Likewise, recent research reports showed that dietary DDGS (20% DM basis) reduced total-tract DM digestibility, but increased metabolizable energy intake and milk yield in dairy cows (61). Similarly, the current meta-analysis shows that including DDGS up to 20% in the diet increases yield grade, and meat color redness (a), and beyond 30%, it shows the reducing effect (62). Likewise, older animals have higher myoglobin concentrations, and the color of the meat may differ according to the age at slaughter (54). Variations in meat color may also be due to breed differences (5). This result suggests that the inclusion of DDGS in the diet has no negative effects on carcass characteristics (19).

Previous studies with dairy cows showed that 20% dietary DDGS improved digestibility, energy intake, and milk yield (35), and moreover, studies have indicated that including up to 60% of DDGS increases nitrogen intake, nitrogen digestibility, and urine nitrogen output, while decreasing nitrogen retention (27). Various levels of supplementation, sheep breeds, and the type of feed ingredients in the basal diet that DDGS replaced in different trials may all have an effect on the amount of N is excreted as a result of dietary DDGS inclusion (35). The results of the meta-analysis show that up to 20% of DDGS in the diet causes greater nitrogen intake and decreased urinary nitrogen production. However, there is a linear rise in urine N loss if the inclusion level exceeds 25% as a result of a quadratic increase in N intake in the diet. These results indicate that addition of DDGS at higher levels may increase the N loss, consequently causing environmental pollution (35).

## Conclusion

Based on data collected from across the literature, dietary DDGS increased performance and carcass weight in meat sheep. Based on the dose–response analysis, the amount of DDGS in the diet should not be more than 20% in order to prevent negative effects on rumen fermentation, nitrogen metabolism, and meat coloring. Although dietary DDGS has been associated with increased NDF and EE digestibility, more research is required to fully understand its effect on DMD. Increased nitrogen intake and decreased urine nitrogen losses were observed when 20% DDGS was included in the diet. The excess bypass protein in DDGS reduced rumen fermentation and increased fecal nitrogen losses in sheep. However, a 20% inclusion rate in the diet of DDGS increased carcass yield and meat color. Due to the limited data, inconsistent responses were observed among comparisons conducted with crossbreeds and purebred animals. In conclusion, this meta-analysis supports the notion that DDGS at low concentrations can enhance sheep performance, improve nitrogen metabolism, and increase carcass yield and meat color.

## Data availability statement

The raw data supporting the conclusions of this article will be made available by the authors, without undue reservation.

## Author contributions

AP-C: conceived and supervised the study and acquired funding. SC and AP-C: inputs to data analysis. SC, TT, ZE-R, IO, and AP-C: methodology, conducted the experiment, and wrote the full paper. All authors contributed to the article and approved the submitted version.

## Funding

This research is funded by USDA grant number 1022336.

## Conflict of interest

The authors declare that the research was conducted in the absence of any commercial or financial relationships that could be construed as a potential conflict of interest.

## Publisher’s note

All claims expressed in this article are solely those of the authors and do not necessarily represent those of their affiliated organizations, or those of the publisher, the editors and the reviewers. Any product that may be evaluated in this article, or claim that may be made by its manufacturer, is not guaranteed or endorsed by the publisher.

## Footnotes

^1^ https://cran.r-project.org/web/packages/robumeta/robumeta.pdf

^2^ https://cran.r-project.org/web/packages/metafor

## References

[1] Karaca S Erdogan S Güney M Çakmakçı C Sarıbey M Kor A. Does the length of time dried distillers' grain with solubles substitution for soybean meal affect physiological indicators and meat quality in finishing lambs? Anim Sci J. (2021) 92:e13561. doi: 10.1111/asj.13561, PMID: 34018642

[2] Abudabos AM Abdelrahman MM Alatiyat RM Aljumaah MR Al Jassim R Stanley D. Effect of dietary inclusion of graded levels of distillers dried grains with solubles on the performance, blood profile and rumen microbiota of Najdi lambs. J Heliyon. (2021) 7:e05683. doi: 10.1016/j.heliyon.2020.e05683, PMID: 33553711 PMC7848638

[3] Chen J Niu X Li F Li F Guo L. Replacing soybean meal with distillers dried grains with solubles plus rumen-protected lysine and methionine: effects on growth performance, nutrients digestion, rumen fermentation, and serum parameters in Hu sheep. Animals. (2021) 11:11. doi: 10.3390/ani11082428, PMID: 34438885 PMC8388632

[4] Quadros DG Kerth CR. Replacing cottonseed meal and sorghum with dried distillers’ grains with solubles enhances the growth performance, carcass traits, and meat quality of feedlot lambs. Transl Anim Sci. (2022) 6:txac040. doi: 10.1093/tas/txac040, PMID: 35669947 PMC9159527

[5] Hatamleh SM Obeidat BS. Growth performance and carcass traits responses to dried distillers’ grain with solubles feeding of growing Awassi ram lambs. Animals. (2019) 9:9. doi: 10.3390/ani9110954, PMID: 31718068 PMC6912553

[6] Curzaynz-Leyva KR Bárcena-Gama JR Sánchez-del Real C Escobar-España JC Rivas-Martínez MI Santillán-Gómez EA. Effect of dried distillers grains (DDGS) on diet digestibility, growth performance, and carcass characteristics in creole wool lambs fed finishing diets. S Afr J Anim Sci. (2019) 49:56–62. doi: 10.4314/sajas.v49i1.7

[7] Reddy PPR Chakrawarthi MK Reddy DM Venkateswarlu S Reddy JB Babu PR. Effect of dried distillers’ grain with solubles as a replacer of peanut cake for sheep fed on low quality forage. Trop Anim Health Prod. (2021) 53:374. doi: 10.1007/s11250-021-02821-0, PMID: 34181100

[8] Ghoneem WM Mahmoud AEM. Impact of dried distillers grains with solubles on productive performance of Barki lambs. Egypt J Nutr Feeds. (2016) 19:30–9.

[9] Reddy PRK Lakshmi RKS Raju J Kishore KR Kumar CA. Cornell net carbohydrate and protein system (CNCPS) fractionations and in-vitro nutrient digestibility of corn dried distiller grains with Solubles (DDGS) from various ethanol plants in Andhra Pradesh. Int J Livest Res. (2017) 7:164–71. doi: 10.5455/ijlr.20170209071816

[10] Alshdaifat SN Obeidat BS. The impact of feeding corn dried distillers grains with solubles on milk yield and composition in lactating Awassi ewes and digestibility and N partitioning in Awassi ewe lambs. Ital J Anim Sci. (2019) 18:522–9. doi: 10.1080/1828051X.2018.1547126

[11] Griffin WA Bremer VR Klopfenstein TJ Stalker LA Lomas LW Moyer JL. A meta-analysis evaluation of supplementing dried distillers grains plus solubles to cattle consuming forage-based diets 1. Prof Anim Sci. (2012) 28:306–12. doi: 10.15232/S1080-7446(15)30360-0

[12] Hollmann M Allen MS Beede DK. Dietary protein quality and quantity affect lactational responses to corn distillers grains: a meta-analysis. J Dairy Sci. (2011) 94:2022–30. doi: 10.3168/jds.2010-3712, PMID: 21426993

[13] Curzaynz-Leyva KR Bárcena-Gama JR Hernández-Sánchez D Crosby-Galván MM Escobar-España JC Santillán-Gómez EA. Corn based-diets containing corn dried distillers grains with solubles on performance, ruminal fermentation, in vitro methane emissions, carcass and meat quality of lambs. Asian J Res Anim Vet Sci. (2020) 5:30–4.

[14] Oliveira AS Weinberg ZG Ogunade IM Cervantes AAP Arriola KG Jiang Y. Meta-analysis of effects of inoculation with homofermentative and facultative heterofermentative lactic acid bacteria on silage fermentation, aerobic stability, and the performance of dairy cows. J Dairy Sci. (2017) 100:4587–603. doi: 10.3168/jds.2016-11815, PMID: 28342607

[15] Arriola KG Oliveira AS Jiang Y Kim D Silva HM Kim SC. Meta-analysis of effects of inoculation with *Lactobacillus buchneri*, with or without other bacteria, on silage fermentation, aerobic stability, and performance of dairy cows. J Dairy Sci. (2021) 104:7653–70. doi: 10.3168/jds.2020-19647, PMID: 33814134

[16] Moher D Liberati A Tetzlaff J Altman DG Altman D Antes G. Preferred reporting items for systematic reviews and meta-analyses: the PRISMA statement. PLoS Med. (2009) 6:e1000097. doi: 10.1371/journal.pmed.1000097, PMID: 19621072 PMC2707599

[17] Archibeque SL Freetly HC Ferrell CL. Feeding distillers grains supplements to improve amino acid nutriture of lambs consuming moderate-quality forages. J Anim Sci. (2008) 86:691–701. doi: 10.2527/jas.2007-0139, PMID: 18073290

[18] Crane AR Redden RR Crouse MS Kirsch JD Borowicz PP Held JE. Influence of distiller's dried grains with solubles on ram lamb growth and reproductive traits. J Anim Sci. (2018) 96:1484–94. doi: 10.1093/jas/sky031, PMID: 29471403 PMC6140920

[19] Van Emon ML Vonnahme KA Berg PT Redden RR Thompson MM Kirsch JD. Influence of level of dried distillers grains with solubles on feedlot performance, carcass characteristics, serum testosterone concentrations, and spermatozoa motility and concentration of growing rams 1. J Anim Sci. (2013) 91:5821–8. doi: 10.2527/jas2013-6369, PMID: 24146156

[20] Crane AR Redden RR Swanson KC Howard BM Frick TJ Maddock-Carlin KR. Effects of dried distiller’s grains and lasalocid inclusion on feedlot lamb growth, carcass traits, nutrient digestibility, ruminal fluid volatile fatty acid concentrations, and ruminal hydrogen sulfide concentration. J Anim Sci. (2017) 95:3198–205. doi: 10.2527/jas2017.1369 28727092

[21] Castro-Pérez BI Estrada-Angulo A Ríos FG Dávila-Ramos H Robles-Estrada JC Contreras-Pérez G. Effects of replacing partially dry-rolled corn and soybean meal with different levels of dried distillers grains with solubles on growth performance, dietary energetics, and carcass characteristics in hairy lambs fed a finishing diet. Small Rumin Res. (2014) 119:8–15. doi: 10.1016/j.smallrumres.2014.03.007

[22] Gabr AA El-Shinnawy MM Makled EH Tag El-Din NTH. Effect of including dried distillers grain with soluble in growing lambs diet on digestibility, some rumen parameters, blood constituents, and performance. J Anim Poult Prod. (2010) 1:251–64. doi: 10.21608/JAPPMU.2010.86221

[23] Lundy EL Loy DD Hansen SL. Influence of distillers grains resulting from a cellulosic ethanol process utilizing corn kernel fiber on nutrient digestibility of lambs and steer feedlot performance. J Anim Sci. (2015) 93:2265–74. doi: 10.2527/jas.2014-8572, PMID: 26020323

[24] Lodge SL Stock RA Klopfenstein TJ Shain DH Herold DW. Evaluation of wet distillers composite for finishing ruminants. J Anim Sci. (1997) 75:44–50. doi: 10.2527/1997.75144x, PMID: 9027547

[25] Huls T Bartosh A Daniel J Zelinsky R Held J Wertz-Lutz A. Efficacy of dried Distiller's grains with Solubles as a replacement for soybean meal and a portion of the corn in a finishing lamb Diet1. Sheep Goat Res J. (2006) 21:30–4.

[26] Obeidat BS. Influence of corn-dried distiller’s grain with solubles on growth performance and blood metabolites of Awassi lambs offered a concentrate diet. Ital J Anim Sci. (2018) 17:636–42. doi: 10.1080/1828051X.2017.1404946

[27] Aloueedat MK Obeidat BS Awawdeh MS. Effects of partial replacement of conventional with alternative feeds on nutrient intake, digestibility, milk yield and composition of Awassi ewes and lambs. Animals. (2019) 9:9. doi: 10.3390/ani9090684, PMID: 31540141 PMC6769483

[28] Mceachern J Whitney T Scott C Lupton C Salisbury M. Substituting distillers dried grains for cottonseed meal in lamb-finishing diets: growth, wool characteristics, and serum NEFA, urea N, and IGF-1 concentrations. Sheep Goat Res J. (2009) 24:32–4.

[29] Neville BW Lardy GP Karges KK Kirschten LA Schauer CS. S intake, excretion, and ruminal hydrogen sulfide concentrations in lambs fed increasing concentrations of distillers dried grains with solubles. Sheep Goat Res J. (2011) 26:13–9.

[30] Schauer CS Stamm MM Maddock TD Berg PB. Feeding of DDGS in lamb rations Feeding dried distillers grains with solubles as 60 percent of lamb finishing rations results in acceptable performance and carcass quality. Sheep Goat Res J. (2008) 23:15–9.

[31] Avila-Stagno J Chaves AV He ML McAllister TA. Increasing concentrations of wheat dry distillers’ grains with solubles in iso-nitrogenous finishing diets reduce lamb performance. Small Rumin Res. (2013) 114:10–9. doi: 10.1016/j.smallrumres.2013.05.003

[32] O’Hara AS Tanner A McAllister TA Gibb DJ Van Herk F Chaves AV. Effect of low and high oil corn distillers’ grain on rumen fermentation, growth performance and carcass characteristics of lambs. Anim Prod Sci. (2011) 51:708–16. doi: 10.1071/AN11023

[33] Graham AS Jonas E Tanner A Avila-Stagno J Bush BD Chaves AV. Effects of replacing rolled barley grain with wheat dry distillers' grains with solubles in merino sheep rations. Acta Agric Scand A Anim Sci. (2013) 63:101–10. doi: 10.1080/09064702.2013.824020

[34] Moyo R Niekerk WAV Hassen A Du Toit CJL Coertze R Adejoro FA. Nutritional quality of wet distillers’ grains co–ensiled with whole–plant maize and its feeding value for lambs. Sci Agric. (2021) 79:79. doi: 10.1590/1678-992X-2020-0122

[35] Shen J Chen Y Moraes LE Yu Z Zhu W. Effects of dietary protein sources and nisin on rumen fermentation, nutrient digestion, plasma metabolites, nitrogen utilization, and growth performance in growing lambs. J Anim Sci. (2018) 96:1929–38. doi: 10.1093/jas/sky086, PMID: 29514293 PMC6140948

[36] Abdelrahim GM Khatiwada J Gurung NK. Effects of dried distillers grains with solubles on performance and carcass characteristics of lamb. J Anim Sci Technol. (2014) 1:25–30. doi: 10.5147/jart.2014.0203

[37] Castro-Pérez BI Garzón-Proaño JS López-Soto MA Barreras A González VM Plascencia A. Effects of replacing dry-rolled corn with increasing levels of corn dried distillers grains with solubles on characteristics of digestion, microbial protein synthesis and digestible energy of diet in hair lambs fed high-concentrate diets. Asian Australas J Anim Sci. (2013) 26:1152–9. doi: 10.5713/ajas.2013.13054, PMID: 25049896 PMC4093229

[38] De Evan T Cabezas A Fuente J Carro MD. Feeding agroindustrial byproducts to light lambs: influence on growth performance, diet digestibility, nitrogen balance, ruminal fermentation, and plasma metabolites. Animals. (2020) 10:10. doi: 10.3390/ani10040600, PMID: 32244765 PMC7222727

[39] Kawęcka A Sosin-Bzducha E Puchała M Sikora J. Effect of maize DDGS addition on carcass and meat quality of lambs of native sheep breed. J Appl Anim Res. (2018) 46:301–5. doi: 10.1080/09712119.2017.1299014

[40] Şahin T Karadagoglu KO Elmalı D Kaya I. Effects of dietary supplementation with distiller dried grain with solubles in growing lambs on growth, nutrient digestibility and rumen parameters. Rev Med Vet. (2013) 164:173–8.

[41] Zelinsky RD Wertz-Lutz AE Held JE. *Effects of increasing dietary energy density by replacing Hay with Soyhulls (SH) and dried distillers grains with Solubles (DDGS) on nutrient digestibility and rumen physiology*. South Dakota State University Sheep Research Report, p. 4. (2014).

[42] Felix TL Zerby HN Moeller SJ Loerch SC. Effects of increasing dried distillers grains with solubles on performance, carcass characteristics, and digestibility of feedlot lambs. J Anim Sci. (2012) 90:1356–63. doi: 10.2527/jas.2011-4373, PMID: 22147466

[43] Tipton E. Small sample adjustments for robust variance estimation with meta-regression. Psychol Methods. (2015) 20:375–93. doi: 10.1037/met0000011, PMID: 24773356

[44] Higgins JPT. Commentary: heterogeneity in meta-analysis should be expected and appropriately quantified. Int J Epidemiol. (2008) 37:1158–60. doi: 10.1093/ije/dyn204, PMID: 18832388

[45] Lean IJ De Ondarza MB Sniffen CJ Santos JEP Griswold KE. Meta-analysis to predict the effects of metabolizable amino acids on dairy cattle performance. J Dairy Sci. (2018) 101:340–64. doi: 10.3168/jds.2016-12493, PMID: 29128215

[46] Hedges LV Tipton E Johnson MC. Robust variance estimation in meta-regression with dependent effect size estimates. Res Synth Methods. (2010) 1:39–65. doi: 10.1002/jrsm.5, PMID: 26056092

[47] Fisher Z Tipton E Zhipeng H Fisher MZ. *Package Robumeta*. R package version (2017).

[48] Egger M Smith GD Schneider M Minder C. Bias in meta-analysis detected by a simple, graphical test. BMJ. (1997) 315:629–34. doi: 10.1136/bmj.315.7109.629, PMID: 9310563 PMC2127453

[49] Pech-Cervantes AA Ferraretto FL Ogunade IM. Meta-analysis of the effects of the dietary application of exogenous alpha-amylase preparations on performance, nutrient digestibility, and rumen fermentation of lactating dairy cows. J Anim Sci. (2022) 100:skac189r. doi: 10.1093/jas/skac189, PMID: 35589551 PMC9387633

[50] Viechtbauer W. Conducting meta-analyses in R with the metafor. J Stat Softw. (2010) 36:1–48. doi: 10.18637/jss.v036.i03

[51] Greenland S. Dose-response and trend analysis in epidemiology: alternatives to categorical analysis. Epidemiology. (1995) 6:356–65. doi: 10.1097/00001648-199507000-00005 7548341

[52] Greenland S Longnecker MP. Methods for trend estimation from summarized dose-response data, with applications to meta-analysis. Am J Epidemiol. (1992) 135:1301–9. doi: 10.1093/oxfordjournals.aje.a116237, PMID: 1626547

[53] Fisher Z Tipton E Zhipeng H. *Robumeta: robust variance meta-regression*. R package version 16 Package ‘robumeta’. (2015).

[54] Gomes HFB de Castro AA Emmert AQ Marques RO Gonçalves HC Brito EP. Substitution of soybean meal with dried distillery grains on performance and carcass quality of feedlot lambs. Semin Cienc Agrar. (2020) 41:2259–72. doi: 10.5433/1679-0359.2020v41n5Supl1p2259

[55] Luebbe MK Patterson JM Jenkins KH Buttrey EK Davis TC Clark BE. Wet distillers grains plus solubles concentration in steam-flaked-corn-based diets: effects on feedlot cattle performance, carcass characteristics, nutrient digestibility, and ruminal fermentation characteristics. J Anim Sci. (2012) 90:1589–602. doi: 10.2527/jas.2011-4567, PMID: 22147473

[56] Mavrogenis AP. Comparative performance of purebred and crossbred sheep in three different production systems. In: Gabiña D. (ed.), Bodin L. (ed.). Data collection and definition of objectives in sheep and goat breeding programmes: New prospects. Zaragoza: CIHEAM, (1997). p. 181–185.

[57] Li J Vasanthan T Gao J Naguleswaran S Zijlstra RT Bressler DC. Resistant starch escaped from ethanol production: evidence from confocal laser scanning microscopy of distiller’s dried grains with solubles (DDGS). Cereal Chem. (2014) 91:130–8. doi: 10.1094/CCHEM-05-13-0087-R

[58] Vries SD Gerrits WJ Kabel MA Vasanthan T Zijlstra RT. β-Glucans and resistant starch Alter the fermentation of recalcitrant fibers in growing pigs. PLoS One. (2016) 11:e0167624. doi: 10.1371/journal.pone.0167624, PMID: 27911928 PMC5135129

[59] Regassa A Nyachoti CM. Application of resistant starch in swine and poultry diets with particular reference to gut health and function. Anim Nutr. (2018) 4:305–10. doi: 10.1016/j.aninu.2018.04.001, PMID: 30175259 PMC6116817

[60] De Boever JL Blok MC Millet S Vanacker J De Campeneere S. The energy and protein value of wheat, maize and blend DDGS for cattle and evaluation of prediction methods. Animal. (2014) 8:1839–50. doi: 10.1017/S1751731114001815, PMID: 25068803

[61] Judy JV Bachman GC Brown-Brandl TM Fernando SC Hales KE Miller PS. Reducing methane production with corn oil and calcium sulfate: responses on whole-animal energy and nitrogen balance in dairy cattle. J Dairy Sci. (2019) 102:2054–67. doi: 10.3168/jds.2018-14567, PMID: 30612805

[62] Van Emon ML Gunn PJ Neary MK Lemenager RP Schultz AF Lake SL. Effects of added protein and dietary fat on lamb performance and carcass characteristics when fed differing levels of dried distiller’s grains with solubles. Small Rumin Res. (2012) 103:164–8. doi: 10.1016/j.smallrumres.2011.09.002

