# Supplementary material for: Effects of dietary inclusion of dry distillers grains with solubles on performance, carcass characteristics, and nitrogen metabolism in meat sheep: a meta-analysis
Source: Front Vet Sci. 2023 Jun 20;10:1141068. doi: 10.3389/fvets.2023.1141068 (PMC10318436; doi:10.3389/fvets.2023.1141068)
Supplement: Supplementary file 1 [file Image_1.pdf]

Effects of dietary inclusion of dry distillers grains with solubles (DDGS) on performance, carcass characteristics, and nitrogen metabolism in meat sheep: A Meta-analysis

S. C. Chelkapally[1], T. H. Terrill[1], Z. M. Estrada-Reyes[2], I. M. Ogunade[3], and A. A. Pech-Cervantes[1]

[1]Agricultural Research Station, Fort Valley State University, Fort Valley 31030, GA, USA

[2]Department of Animal Science, North Carolina A&T State University, Greensboro, NC 27411, USA

[3]Division of Animal and Nutritional Science, West Virginia University, Morgantown, WV 26505

*Corresponding author = andres.pechcervantes@fvsu.edu

Supplementary file
Appendix 1.

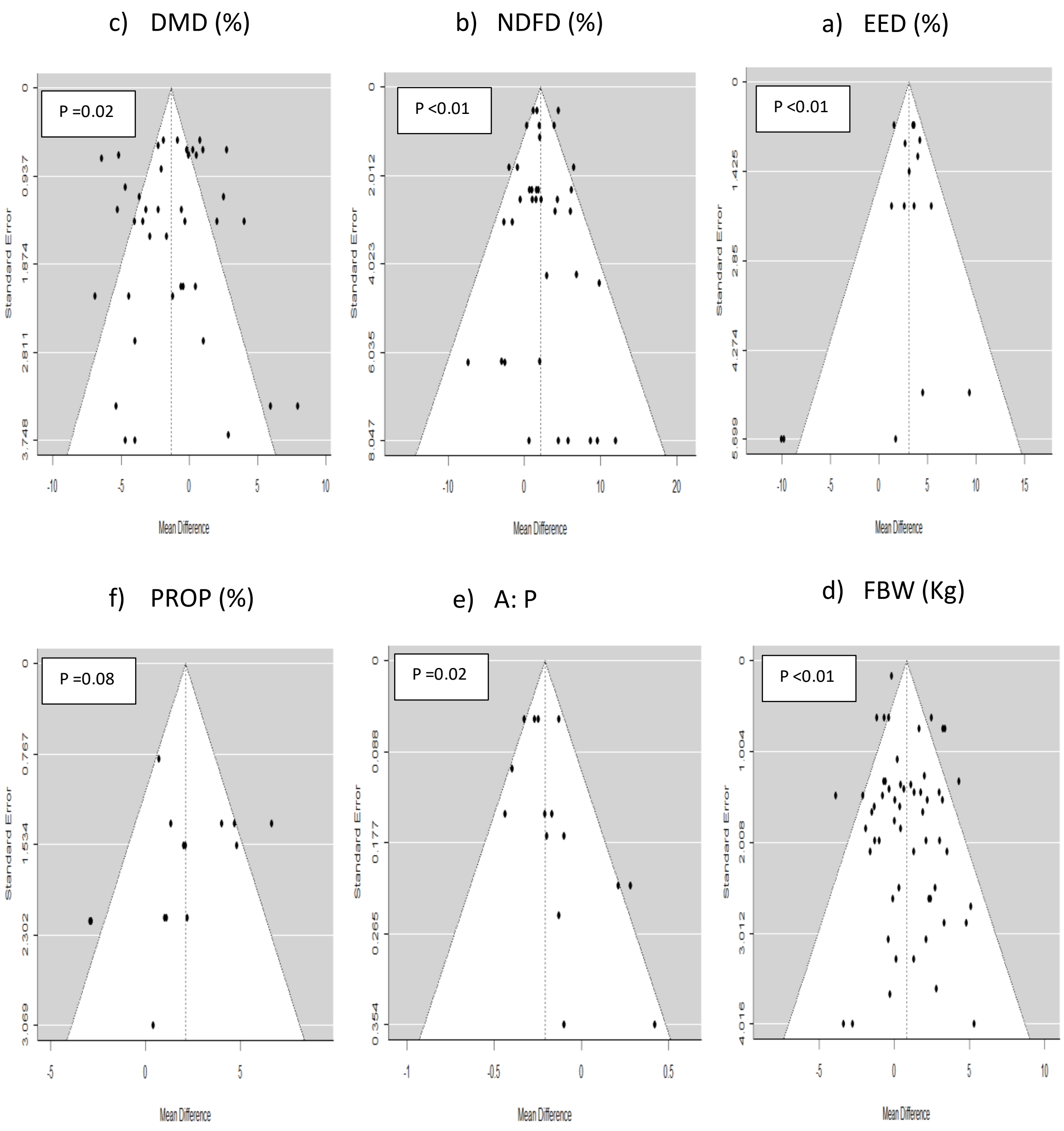

Funnel plots showing the effect of inclusion of DDGS on a) Total- tract dry matter digestibility (N= 38), b) Total- neutral detergent fiber digestibility (N =34), c) Total- ether extract digestibility (N =16), d) Propionate (N=14), e) Acetate: Propionate ratio (N = 21) and, Final body weight (N=57). The horizontal line indicates the raw mean difference (RMD) and the vertical line standard error (SEM). The P-values correspond to the funnel plot asymmetry test by Egger’s regression method.

Appendix 2.

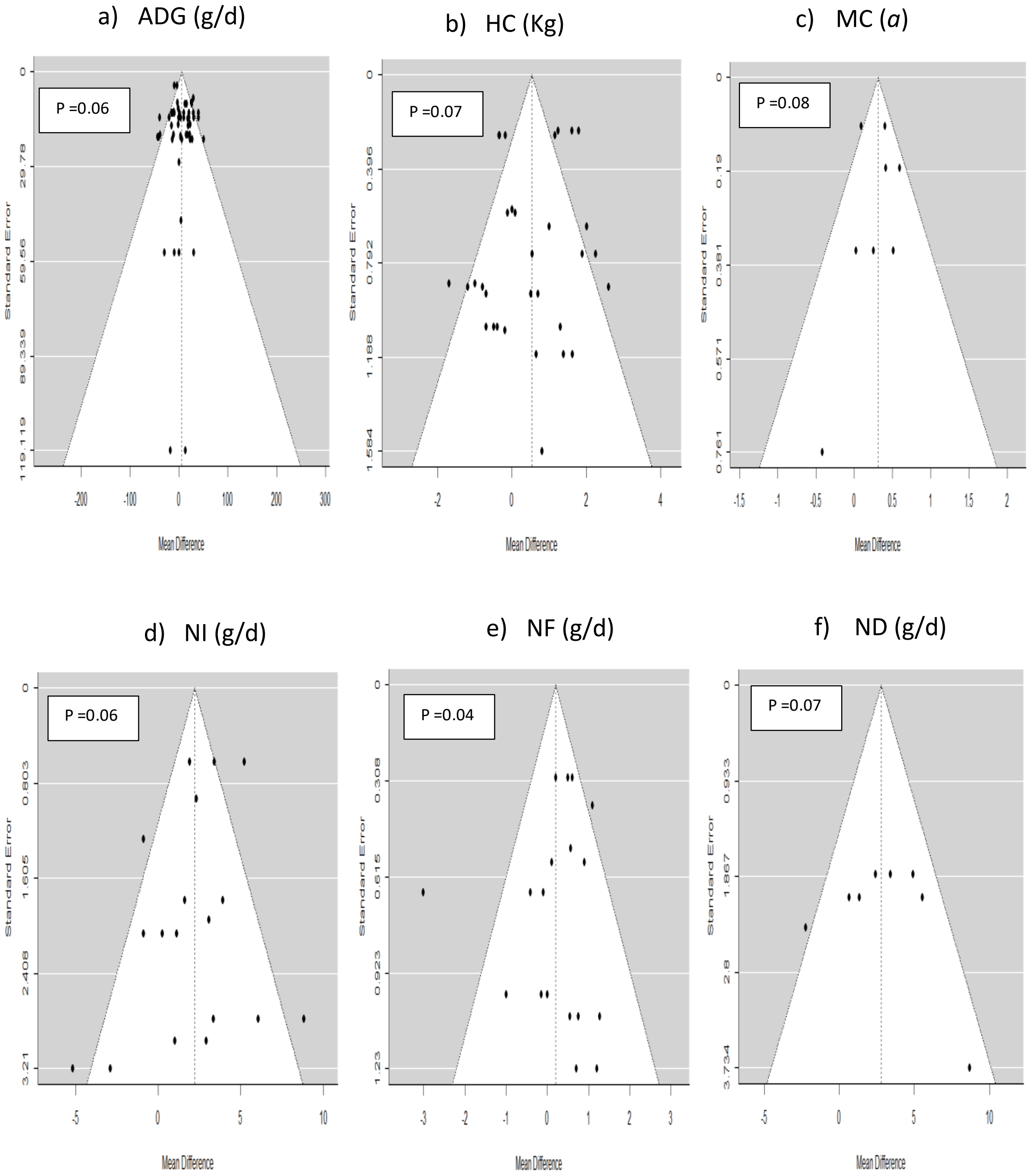

Funnel plots showing the effect of inclusion of DDGS on a) Average body gain (N= 60), b) Hot carcass weight (N =34), c) Muscle color (*a*) redness (N =8), d) Nitrogen intake (N=18), e) Nitrogen feces (N = 19) and, Nitrogen digestibility (N=8). The horizontal line indicates the raw mean difference (RMD) and the vertical line standard error (SEM). The P-values correspond to the funnel plot asymmetry test by Egger's regression method.
